# Morphometric Effects of HO-1 Deficiency and Overexpression in Rat Glomeruli and Podocytes

**Published:** 2021-06-28

**Authors:** Kelsey Wilson, Maria G Detsika, Elpida Poulaki, Harikleia Gakiopoulou, Elias A Lianos

**Affiliations:** 1 Veterans Affairs Medical Center and Virginia Tech., Carilion School of Medicine, USA; 2 First Department of Critical Care Medicine & Pulmonary Services, Evangelismos Hospital, National and Kapodistrian University Athens, Greece; 3 First Department of Pathology, School of Medicine, National and Kapodistrian University of Athens, Greece

## Podocytes in Glomerular Injury

Of the various cells comprising the glomerulus (endothelial, mesangial, resident macrophages, and visceral glomerular epithelial cells also known as podocytes), the latter are most vulnerable to various forms of glomerular injury (Diabetes, Hypertension, immune-mediated, etc.) that frequently progress to end-stage kidney disease (ESKD). This is due to their non-replicative, terminally differentiated highly specialized nature. Specifically, they exhibit a high rate of vesicular traffic as evidenced by multiple coated vesicles and coated pits along their basolateral domain and have a high capacity for protein synthesis and posttranslational modifications because of a well-developed endoplasmic reticulum and a large Golgi apparatus [1]. Podocyte depletion consequent to injury is a well-established mechanism underlying glomerular scarring (sclerosis) while progressive glomerulosclerosis leads to ESKD [2]. It is, therefore, not surprising that strategies assessing ways to protect podocytes against injury are being explored.

## Podocyte Protection Strategies: Role of Heme Oxygenase (HO)

Podocyte protection strategies have recently aimed at various molecular targets with therapeutic potential. These include podocyte proteins that are critical for maintaining the structural integrity of the slit diaphragm and have also been linked to signalling pathways [3], inhibition of the podocyte Angiotensin II receptor [4], and drugs currently in clinical practice shown to preserve the actin cytoskeleton of podocytes. These include Renin-Angiotensin System inhibitors/antagonists, glucocorticoids, cyclosporin A, monoclonal antibodies (rituximab), fusion proteins interfering with the immune activity of T cells (abatacept), and antineoplastic agents (dasanatinib) [5]. Oxidative stress also plays a role in mediating podocyte injury and glomerulosclerosis. Therefore, strategies aiming to mitigate oxidative stress, for example by augmenting the activity of naturally occurring antioxidant enzymes, have also been explored [6].

Within the past two decades, the inducible isoform of Heme Oxygenase (HO-1), which is the rate-limiting enzyme of heme degradation, has been widely accepted as a renoprotective enzyme based on established antioxidant / cytoprotective effects of the heme degradation products, carbon monoxide (CO) and biliverdin, and on observations that HO-1 induction occurs in a wide spectrum of renal pathologies. These include glomerular, tubular, interstitial, and renovascular disease including hypertension and sickle cell nephropathy [7]. HO (also known as heat shock protein 32) is a microsomal enzyme that attenuates heme-induced cell toxicity by degrading free heme released from heme-containing proteins, including hemoglobin, myoglobin, and cytochromes. In the HO reaction, free heme is degraded to CO and biliverdin utilizing NADPH and molecular oxygen as cofactors. Bilirubin is a free radical scavenger that blocks lipid peroxidation. CO is a vasodilator acting via cyclic guanosine monophosphate (cGMP). Ferrous (Fe^++^) iron, also released from heme during the HO reaction, induces ferritin which is also cytoprotective.

The role of HO-1 in protecting podocytes against injury has been studied mainly in experimental models. In a rat model of hypertension consequent to inhibition of Nitric Oxide (NO) production, treatment with the natural HO inducer heme-arginate enhanced expression of key podocyte structural proteins including nephrin, podocin, podocalyxin, and CD2AP, reduced proteinuria, and improved renal function [8]. Similar beneficial effects of HO-1 induction were described in a rat model of obesity-associated podocytopathy and were attributed to the reduction of pro-inflammatory/oxidative mediators including, macrophage-inflammatory-protein-1α, macrophage-chemoattractant-protein-1, and 8-isoprostane [9]. In a rat model of doxorubicin-induced podocyte injury resulting in nephrotic syndrome, administration of the naturally occurring phenolic compound, curcumin, activated nuclear factor erythroid-2-related factor 2 (Nrf2)-dependent genes and their proteins including heme oxygenase-1 (HO-1), inhibited the pro-inflammatory NF-κB pathway and up-regulated expression of the podocyte structural protein, podocin [10]. In a rat model of diabetes, HO-1 inhibition increased the extent of podocyte apoptosis [11]. In mice with intravascular hemolysis associated with increased levels of plasma hemoglobin (Hb) and heme, podocytes were shown to take up Hb and heme and develop injury characterized by foot process effacement, decreased synaptopodin and nephrin expression, and apoptosis. These pathological effects were augmented in mice lacking the HO-1 up regulator, Nrf2, indicating that HO-1 plays an important role in mitigating the toxicity of podocytes exposed to free heme [12].

Collectively, these studies support a protective effect of podocyte HO-1 induction against various forms of injury. However, administration of HO-1 inducers to achieve increased expression in podocytes may have off-target effects mediated by mechanisms independent of HO-1 induction, and such effects may also account for attenuation of podocyte injury. Moreover, compared to other kidney cells, HO-1 induction in podocytes is limited, inefficient or subject to autoregulation. Thus, earlier studies demonstrated that, in response to systemic administration of HO-1 inducers, there was no detectable increase in HO-1 expression in podocytes [13]. A similar finding was reported following direct podocyte injury using the podocyte-specific toxin, puromycin amino nucleoside, that generates reactive oxygen radicals known to induce HO-1 [14], and in a rat model of immune-mediated glomerular injury in which intraglomerular release of potent HO-1 inducers, including cytokines, occurs [15]. Subsequent in vitro studies using isolated glomeruli with podocyte-targeted HO-1 overexpression demonstrated that the magnitude of HO-1 induction in response to its natural substrate/inducer, heme, is subject to negative modulation raising the possibility that such mechanism could serve to protect against detrimental effects known to occur when HO-1 is overexpressed in certain cell types [16].

## HO-1 Deficiency and Overexpression in Glomerular Podocytes: Morphometric Analysis Studies

To address the direct effect of HO-1 expression in podocytes, Detsika and co-workers used genome-editing approaches to generate rats with either HO-1 deficiency or podocyte-targeted HO-1 overexpression and assessed effects on kidney structure/function. Both HO-1 deficiency and HO-1 overexpression in podocytes had deleterious effects manifested as focal and segmental sclerosing lesions in glomeruli and proteinuria [17,18].

**Figure 1:**
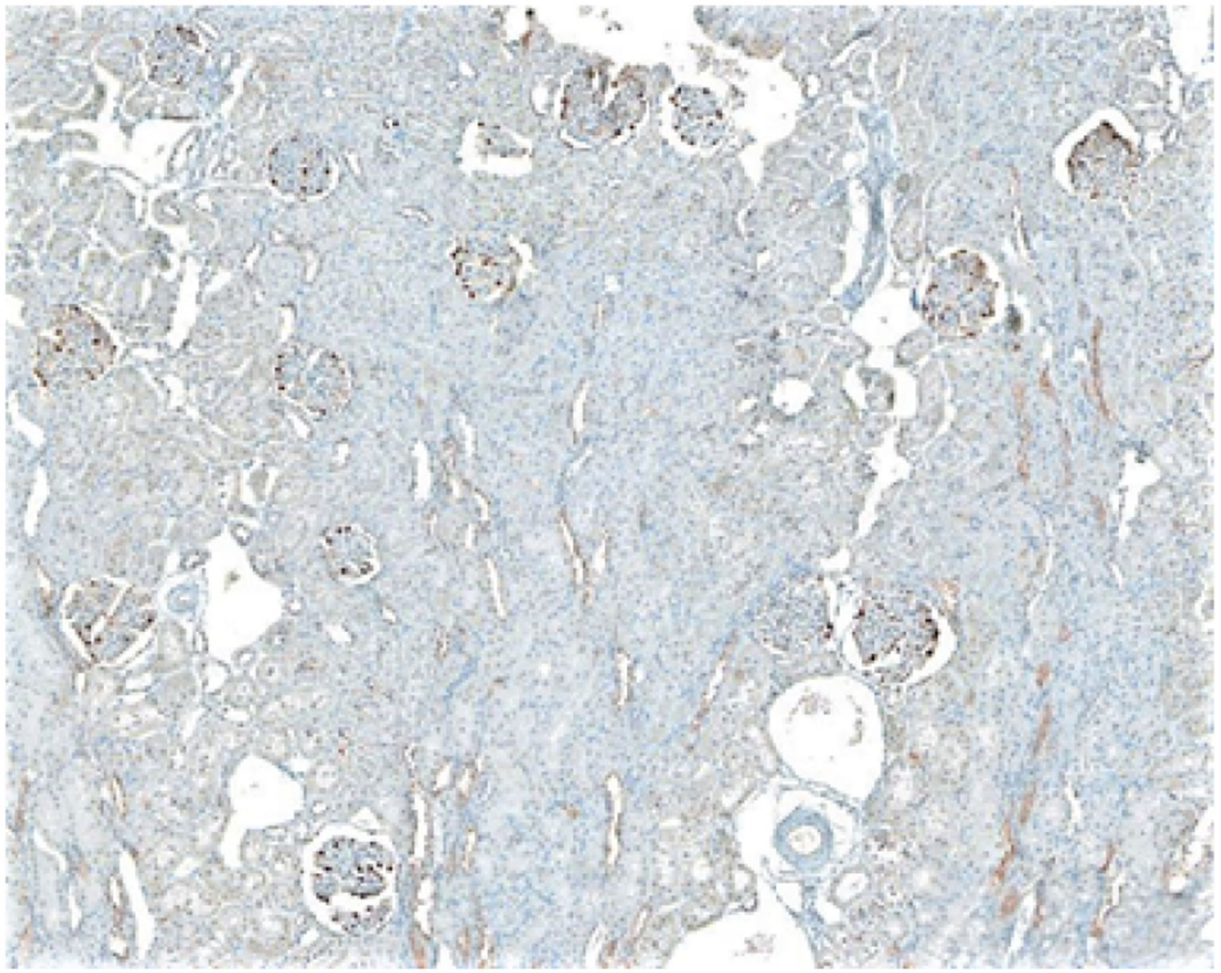
Random 3 μm paraffin section of kidney cortex imaged at 20X magnification with stitching using software of the ImageXpress Pico microscopy system. Clearly shown in all glomeruli are immunoperoxidase stained WT1 positive cells (podocytes).

**Figure 2:**
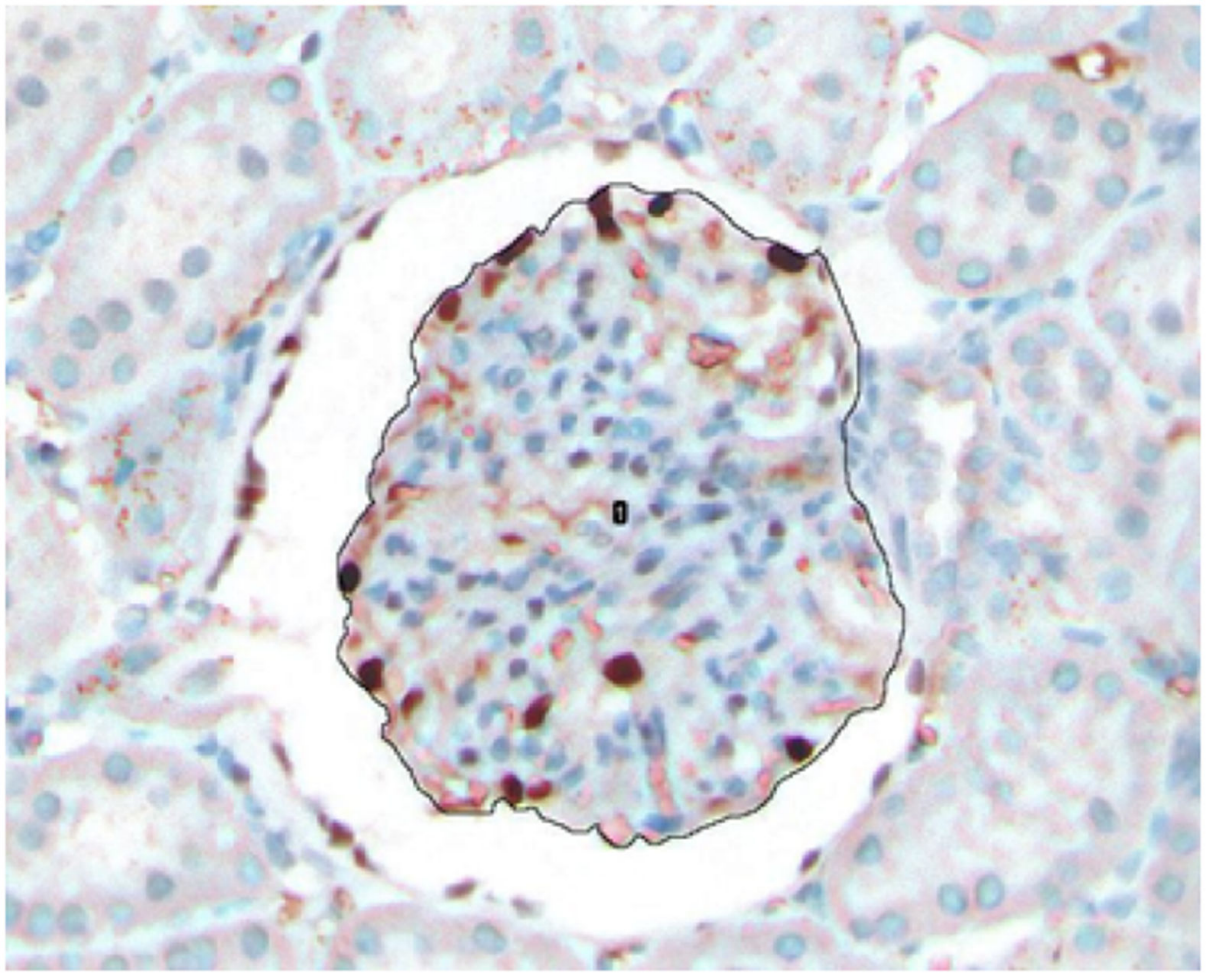
Representative glomerular tuft containing WT1 positive cells (podocytes). Tuft was manually traced using ImageJ software coupled with the ImageXpress Pico microscopy system. In each traced glomerular tuft, surface area, glomerular volume (derived from surface radius) and podocyte number per glomerulus were measured.

Using computational morphometric analysis methods on kidney cortical sections, we measured podocyte number (identified as cell nuclei positive for the podocyte marker, Wilm’s Tumor-WT1- protein) and glomerular volume in all glomeruli present in cortical sections. Random 3μm cortical sections were obtained from paraffin-embedded, formalin-fixed kidney tissue from weight-matched animals in each genotype (Wild-type rats, HO-1 deficient rats, and rats with podocyte-targeted HO-1 overexpression). Sections were stained for WT1 positive cells and examined under an ImageXpress Pico Microscopy system (Molecular Devices, San Jose, CA) at x20 magnification. A representative cortical section is shown in (Figure 1). Images were stitched, enlarged, and the Feret diameter (also known as caliper diameter) of every glomerulus in the section (Figure 1) was measured manually by tracing the glomerular tuft outline (Figure 2) on a computer screen using ImageJ Software 1.26t software (National Institutes of Health; rsb.info.nih.gov/ij).

The ImageJ Program allows each manually traced object to be assigned a number as it is being traced. As the glomerulus can be modelled as a sphere, each glomerular tuft surface area was calculated from the radius value derived after obtaining the caliper diameter (see above). The glomerular volume (V) was then derived using the formula V=4π X r^3^/3, where r=sphere radius, and π=3.14159. The number of WT-1 positive nuclei were counted in each glomerular cross-section. Glomerular tufts included in the morphometric analysis of each were selected using a box-and-whisker plot of the distribution of all glomerular tuft area values obtained in each section. In this plot, the upper and lower quartiles were defined and values that fell outside these quartiles were excluded from the analysis. Figure 2 shows WT1 positive cells (dark brown nuclei) in a glomerulus visualized under high magnification, in which the tuft was traced manually using image J software.

**Figure 3:**
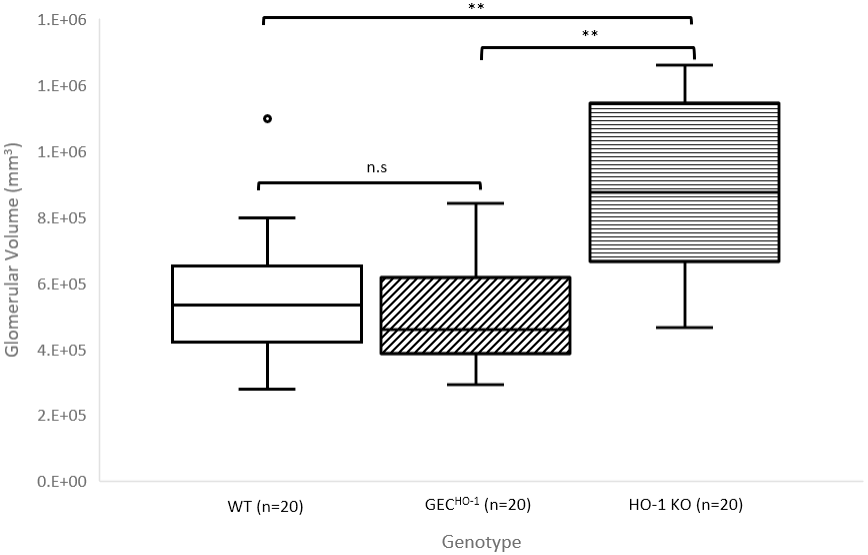
Box-and-whisker plots of glomerular volumes estimated in n=20 glomeruli of cortical sections from three individual weight-matched rats of each genotype [Wild-type (WT), podocyte-targeted HO-1 overexpression (GEC^HO-1^), and HO-1 deficiency (HO-1 knock out (HO-1 KO)]. Asterisks indicate statistical significance (p<0.05). n.s, not significant. Comparisons employed one-way Analysis of Variance (ANOVA). A student’s t-test assuming unequal variances was also completed in each group in which significant differences in glomerular volumes were found. Mean glomerular volume was significantly higher in HO-1 deficient rats.

Glomerular volumes estimated in glomeruli of 3μm cortical sections obtained from weight-matched transgenic rats [Wild-type (WT), podocyte-targeted HO-1 overexpression (GEC^HO-1^), and HO-1 deficient (HO-1 knock out (HO-1 KO)] are shown in (Figure 3). There was a significant increase in mean glomerular volume in the HO-1 deficient compared to the WT and the GEC^HO-1^ rats. There was no statistically significant difference in glomerular volumes between WT and the GEC^HO-1^ rats.

**Figure 4:**
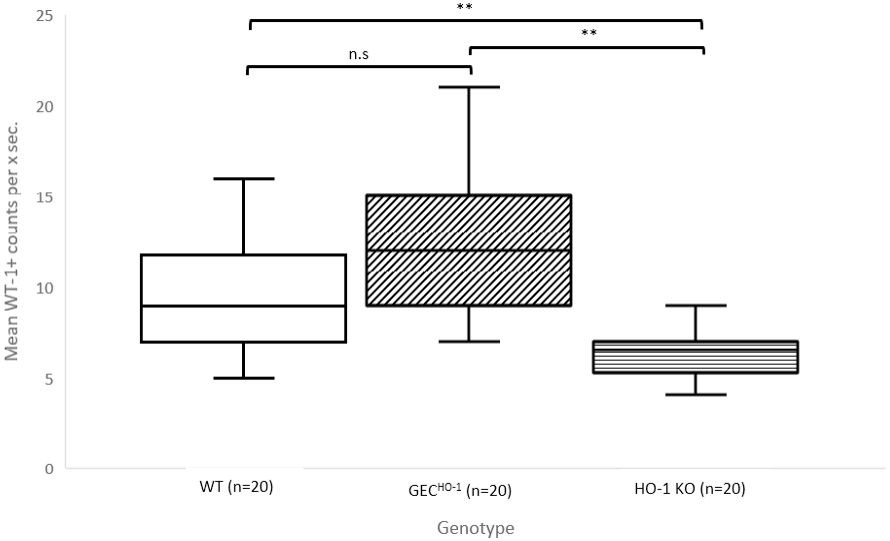
Box-and-whisker plots of Podocyte (WT1 positive nuclei per glomerular section) counts in same glomeruli in which volumes were estimated (shown in Figure 3). Asterisks indicate statistical significance (p<0.05). n.s, not significant. Comparisons employed one-way Analysis of Variance (ANOVA). A student’s t-test assuming unequal variances was also completed in each group in which significant differences in glomerular volumes were found. Mean podocyte number was significantly lower in HO-1 deficient rats.

Podocyte (WT1 positive nuclei per glomerular section) counts in the same cortical sections are shown in (Figure 4). There was a significant decrease in mean podocyte number in the HO-1 deficient (KO HO-1) compared to the WT or the GEC^HO-1^ rat. There was no statistically significant difference in podocyte counts between the WT or the GEC^HO-1^ rat.

The apparent discrepancy between podocyte cell count (decrease) and glomerular volume (increase) in the HO-1 deficient rat is indicative of podocyte loss in edematous glomeruli. This explanation is supported by electron microscopy examination of glomeruli of HO-1 deficient rats showing podocyte edema [17].

## Conclusion

Despite evidence that HO-1 induction affords protection in various forms of kidney injury, targeted induction in podocytes can be detrimental as is constitutive depletion. Therefore, HO-1 is unlikely to be a promising therapeutic target in podocyte protection strategies.
